# Barriers and Motivators to Voluntary Medical Male Circumcision Uptake among Different Age Groups of Men in Zimbabwe: Results from a Mixed Methods Study

**DOI:** 10.1371/journal.pone.0085051

**Published:** 2014-05-06

**Authors:** Karin Hatzold, Webster Mavhu, Phineas Jasi, Kumbirai Chatora, Frances M. Cowan, Noah Taruberekera, Owen Mugurungi, Kim Ahanda, Emmanuel Njeuhmeli

**Affiliations:** 1 Population Services International, Harare, Zimbabwe; 2 Centre for Sexual Health & HIV/AIDS Research (CeSHHAR), Harare, Zimbabwe; 3 Centre for Sexual Health & HIV Research, University College London, London, United Kingdom; 4 Population Services International, Washington, District of Columbia, United States of America; 5 Ministry of Health and Child Welfare, Harare, Zimbabwe; 6 United States Agency for International Development, Washington, District of Columbia, United States of America; Alberta Provincial Laboratory for Public Health/University of Alberta, Canada

## Abstract

**Background:**

We conducted quantitative and qualitative studies to explore barriers and motivating factors to VMMC for HIV prevention, and to assess utilization of existing VMMC communication channels.

**Methods and Findings:**

A population-based survey was conducted with 2350 respondents aged 15–49. Analysis consisted of descriptive statistics and bivariate analysis between circumcision and selected demographics. Logistic regression was used to determine predictors of male circumcision uptake compared to intention to circumcise. Focus group discussions (FGDs) were held with men purposively selected to represent a range of ethnicities. 68% and 53% of female/male respondents, respectively, had heard about VMMC for HIV prevention, mostly through the radio (71%). Among male respondents, 11.3% reported being circumcised and 49% reported willingness to undergo VMMC. Factors which men reported motivated them to undergo VMMC included HIV/STI prevention (44%), improved hygiene (26%), enhanced sexual performance (6%) and cervical cancer prevention for partner (6%). Factors that deterred men from undergoing VMMC included fear of pain (40%), not believing that they were at risk of HIV (18%), lack of partner support (6%). Additionally, there were differences in motivators and barriers by age. FGDs suggested additional barriers including fear of HIV testing, partner refusal, reluctance to abstain from sex and myths and misconceptions.

**Conclusions:**

VMMC demand-creation messages need to be specifically tailored for different ages and should emphasize non-HIV prevention benefits, such as improved hygiene and sexual appeal, and need to address men's fear of pain. Promoting VMMC among women is crucial as they appear to have considerable influence over men's decision to get circumcised.

## Introduction

Thirteen Eastern and Southern African countries are currently scaling-up voluntary medical male circumcision (VMMC) as part of a comprehensive HIV prevention strategy [1], [2], [3]. Mathematical modeling suggests that circumcising 80% of the male population aged 15–49 in these 13 countries by 2015 and sustaining this coverage level thereafter could avert 430,000 new HIV infections by 2015 and 3.36 million HIV infections by 2025 [2], [3]. Among the 13 countries, Zimbabwe has the potential to avert the highest proportion of new HIV infections. It has been estimated that circumcising 1.9 million Zimbabwean men aged 15–49 by 2015 could avert 42% (600,000) of new HIV infections that would have otherwise occurred by 2025 [3]. This same model suggests that prioritizing Zimbabwean males aged 15–29 will lead to the greatest reduction in HIV incidence in the short-term [3]. In addition, the faster the coverage of VMMC can be achieved, the greater the number of infections averted. Prior to the commencement of Zimbabwe's VMMC program in 2009, the country's male circumcision (MC) prevalence (obtained through self-report) was one of the lowest in the Southern African region at 10.3% [4]. Despite rapid scale-up of service provision, uptake of VMMC has been slower than expected with only 170,000 men reached as of September 2013 against a five-year target (2010–2015) of 1.9 million.

Understanding and addressing the barriers and motivators to VMMC uptake to inform effective demand-creation is an urgent priority in Zimbabwe and the region more widely [5]. Generating demand for VMMC represents a social marketing challenge par excellence [6]. It is likely to be particularly challenging to market VMMC in Zimbabwe, a traditionally non-circumcising country, where the connotations of having been circumcised were until recently, largely negative.

Research from other settings suggests that the most common individual barriers to seeking VMMC are: fear of pain associated with the operation and local anesthesia, as well as fear of complications; the lengthy healing and sexual abstinence period; perceived threats to masculinity; and perceived costs [7], [8]. The objective of this study was to explore barriers and motivating factors to VMMC for HIV prevention in Zimbabwe. Resultant findings were used to inform the design of appropriate VMMC communication strategies.

## Methods

We conducted a national, population-based survey in February 2013 with 2350 respondents aged 15–49 years. Assuming 90% of those approached agreed to take part in the survey, this sample size would provide 80% power to detect various predictors of VMMC uptake. The study utilized a multi-stage cluster design. In the first stage, 100 enumeration areas (EAs) were randomly selected using probability proportionate to size (60 rural and 40 urban). In the second stage, households were the sampling unit. A complete listing of households for each of the100 EAs selected was obtained from the Central Statistical Office. The listing included maps for each of the EAs. Simple random sampling was used to select households. In each selected household, a single eligible respondent was interviewed. Respondents were included in the study if they fulfilled the following criteria: aged 15–49 years; being a member of the household for at least 12 months; present at the time scheduled for the interview. In households with more than one eligible respondent, a participant was randomly selected using the Kish grid method. If there was no one at a selected household, two call-backs were done before substitution with the closest adjacent household. If no eligible respondent lived in the household, the closest adjacent household was substituted.

Quantitative data were collected through individual interviews using structured questionnaires conducted by trained field interviewers. The questionnaires were informed by validated questions (e.g., MC knowledge, attitudes) from WHO/UNAIDS [9], behavior change frameworks [10] and the Zimbabwe Demographic and Health Survey (ZDHS) [11]. The questionnaires were programmed using Entryware software and personal digital assistants (PDAs) were used for data collection. Range and consistency checks and skip patterns were programmed into the questionnaire to improve data quality. In addition, trained supervisors monitored data collection in the field and conducted back checks for 10% of all interviews. Bivariate analyses were conducted between circumcision and selected demographic variables. Logistic regression was used to determine predictors of MC compared to intention to circumcise. The outcome variable was defined as males intending to be circumcised versus men already circumcised. The definition of the outcome variable was based on the adoption stairway that builds on behavior change frameworks [10]. We chose to report on comparing intention to circumcise with circumcised because individuals with intention to adopt a behavior are more likely to practice the behavior compared to those in other stages of behavior change.

Predictor variables included perceived availability of VMMC services; self-efficacy (one's belief that one can make the decision to go for VMMC); social support (support from friends to go for VMMC); positive beliefs about MC benefits and safety, and knowledge. Age, education, marital status, socio-economic status and religion were the covariates. The survey data will be uploaded onto a web-based, data sharing platform hosted by Harvard Dataverse Network. This network is available to the public and users can cite and/or request data for reuse.

The qualitative study, conducted in 2010, consisted of focus group discussions (FGDs) held across Zimbabwe with men drawn from seven ethnicities: Shona, Ndebele, Shangaan, Chewa, Xhosa, Venda and Remba. Between June and October 2010, 14 gender-specific FGDs were held with young men (aged 18–24), (n = 7 groups), older men (25–49), (n = 7 groups) in five of Zimbabwe's 10 provinces (Bulawayo, Harare, Mashonaland West, Masvingo and Matebeleland North). Purposive and snowball sampling were employed to ensure that the seven ethnicities (and age groups) were fairly represented in the FGDs. Discussions were conducted in either Shona or Ndebele, Zimbabwe's dominant indigenous languages, also spoken and understood by smaller ethnic groups. Two trained teams of male researchers conducted the FGDs; each team was composed of a facilitator and note-taker. FGDs were guided by a topic guide. Topic guide development was initially informed by a review of MC literature. However, as data collection progressed and additional themes began to emerge, the topic guide was revised. The data collection process was therefore iterative and involved data collection, on-going analysis and topic-guide revisions to test for theme saturation [12], [13].

All discussions were audio-recorded. Audio-recorded qualitative data were transcribed and translated verbatim into English; transcripts were entered into NVivo 8 (QSR International, Melbourne, Australia), a qualitative data storage and retrieval program. Two researchers coded each transcription separately. Discrepancies were resolved by discussion with the study coordinator, who also independently coded all transcripts. Codes were grouped into categories and emerging themes were then identified using thematic analysis [12], [13]. Only qualitative findings on barriers to VMMC are presented in this analysis. These findings were used to augment those from the population-based survey and were illustrated with verbatim quotes.

## Ethical Considerations

All studies obtained ethics approval from the relevant ethics committees including: Medical Research Council of Zimbabwe, the ethics board of Population Services International (PSI) and the University College London ethics committee. All participants provided written informed consent.

## Results

A total of 2350 individuals were interviewed. Nearly half (49.6%, n = 1165) of the survey participants were men, 45% (n = 1058) were aged 15–24 years. The median age was 25 among male respondents and 26 among female respondents. Respondents were mostly Christian (73.3%, n = 1723), married (43.7%, n = 1026) or previously married (12.4%, n = 292), had a secondary education level (76.9%, n = 1807) and were residing in rural areas (60%, n = 1409). Table 1 shows the socio-demographic characteristics of survey participants and main outcome variables.

**Table 1:** Socio-demographic characteristics of survey participants and main outcome variables by gender.

	Subcategory	Men % (n = 1165)	Women % (n = 1185)	Total % (n = 2350)
Sex	Male	-	-	49.6
	Female	-	-	50.4
Age	15–24	22.51	22.51	45.0
	25–49	27.06	27.91	55.0
	Median age	25.0	26.0	25.0
Religion	Traditional	2.1	0.6	2.7
	Roman Catholic	9.0	10.0	19.0
	Pentecostal	13.6	16.7	30.3
	Apostolic sects	8.5	12.8	21.3
	Muslim	0.8	0.6	1.3
	None	11.3	3.2	14.5
	Other	4.3	6.6	10.9
Marital Status	Married/co-habiting	20	23.7	43.7
	Never married	25.6	18.3	43.9
	Widowed	1.3	3.7	5
	Divorced	1.5	2.9	4.4
	Separated	1.3	1.8	3.1
Education	Primary	5.4	8.1	13.5
	Secondary	38.8	38.1	76.9
	University/higher	5.3	3.6	8.9
	None	0.1	0.6	0.7
Place of residence	Rural	29.4	30.5	60.0
	Urban	20.1	19.9	40.0
Ever heard of VMMC as an HIV prevention method	Yes	68.4	53.8	61.0
	No	31.6	46.2	39.0
Willingness to undergo VMMC (oneself/the partner)	Yes	60	71.1	65.6
	No	40	28.9	34.4
Willingness to have one's son undergo VMMC	Yes	75.9	77.9	76.8
	No	24.1	22.1	23.2

### VMMC Knowledge

Sixty-eight percent (n = 797) of the interviewed men and 53.8% (n = 637) of women had heard about VMMC as an HIV prevention intervention. Among male respondents there was no difference in knowledge by age; 82.3% of male respondents who had heard of VMMC knew that it reduced risk of HIV acquisition (84.5% aged 18–24; 80.8% aged 25–49). Most (87.6%) knew that VMMC can protect against sexually transmitted infections, 86.1% knew that VMMC improves penile hygiene and 67.6% knew about its protective effect on cervical cancer in the female partner. The majority (89.1%) understood that VMMC is only partially protective for HIV acquisition and that circumcised men still need to use other HIV prevention methods post circumcision. Nevertheless, 22.6% of the male respondents who had heard about VMMC believed that a circumcised man does not need to use a condom to prevent HIV acquisition and 55.7% believed that HIV-positive individuals should be circumcised (Table 2).

**Table 2:** VMMC knowledge among males aged 15–49 who had heard about VMMC.

VMMC knowledge	15–24 (n = 323)	25–49 (n = 474)	Total (n = 797)	Significance
VMMC reduces risk of HIV acquisition	84.6	80.8	82.3	P = 0.117
VMMC protects against STI	88.5	86.9	87.9	P = 0.495
VMMC improves penile hygiene	84.5	87.2	86.1	P = 0.296
VMMC prevents cervical cancer in women	67.5	67.7	67.6	P = 0.946
Circumcised men still need to use other methods such as condoms and sexual partner reduction	92.0	88.4	89.1	P = 0.103
Once circumcised, a man no longer has to use condoms to prevent HIV	22.9	22.4	22.6	P = 0.856
HIV positive individuals should be circumcised	55.7	55.7	55.7	P = 0.993

### Attitudes Towards VMMC

Among the female respondents who had heard about VMMC, 71.1% reported being supportive of their male partner being circumcised (Table 1) and the majority of the male respondents were either already circumcised 11% (n = 90) or were intending to go for VMMC 49% (n = 389), while 40% (n = 318) reported that they were not interested in getting circumcised. Among both female and male respondents, 76.8% reported willingness to have their son circumcised. When asked whether the male interviewee would recommend VMMC to his peers, 75% of those who had heard about VMMC answered “yes,” with significantly more “yes” respondents from the younger age group than from the older age group (78.6% versus 72.6%, p<0.05) respectively.

### Sources of VMMC Information

The majority (71.4%) of the male respondents cited the radio as the source of information about VMMC (Table 3). Television was the second most frequently mentioned communication channel (40.4%), followed by newspaper (28.9%), billboards (22.2%) and posters (22.2%). Among the interpersonal communication channels, the majority of respondents cited the health and community worker as the primary source of information (28.7%), followed by peers, friends and relatives (26.2%), small group discussions (7.3%), road shows organized by PSI (5.6%), door-to-door visits by community mobilizers (5.2%) and community drama (3.4%).

**Table 3:** Sources of information about VMMC among males aged 15–49.

Sources of information about VMMC	% (n = 1165)
Radio	71.4
Television	40.4
Workplace	11.6
Newspaper/magazine	28.9
Posters	22.2
Billboards	22.2
Health/community worker	28.7
Counselor	8.4
Friends/relatives	26.2
Leaflets/brochures	6.6
Road shows	5.6
Drama/theater	3.4
Door to door	5.2
Small group discussions	7.3
Individual discussions	5.6
Others	10

### Motivating Factors for VMMC Uptake

Table 4 describes the motivating factors for VMMC uptake by age group. Among men in the survey who reported that they were willing to be circumcised, 93.8% reported they were motivated to undergo VMMC to prevent HIV/STIs, 56% for hygiene purposes, 13.4% to enhance sexual performance, 13.1% to prevent cervical cancer in their partner, to set a good example for their community (11.1%) or children (7.5%), as well as to please their female partner (8%). Significantly more men from the older age group cited “improved hygiene” as a motivating factor (62.3% vs. 49.2%, p = 0.01). Older men were also more likely to be motivated by “improved sexual performance” than younger men (18.1% vs. 8.1%, p = 0.004) and setting a good example for the community (14.2% vs. 7.6%, p = 0.037).

**Table 4:** Motivating factors for VMMC by age among males 15–49 year olds willing to be circumcised.

Motivating factor	15–24 years (n = 185)	25–49 years (n = 204)	Total (n = 389)	Significance
HIV prevention	95.1	92.6	93.8	P = 0.308
Personal hygiene	49.2	62.3	56.0	P = 0.01
Improve sexual performance	8.1	18.1	13.4	P = 0.004
Prevent cervical cancer in my partner	10.3	15.7	13.1	P = 0.114
Set a good example for my community	7.6	14.2	11.1	P = 0.037
To please my partner	6.5	9.3	8.0	P = 0.304
Set good example for my children	4.9	9.8	7.5	P = 0.064
Followed my friends	2.2	5.4	3.9	P = 0.098
My partner told me to	2.7	4.4	3.6	P = 0.366
My mother told me to	2.7	1	1.8	P = 0.202

### Barriers to VMMC Uptake: Quantitative Findings

Table 5 describes the barriers to VMMC uptake by age group among men who reported that they were not willing to be circumcised. The most frequently cited barrier to circumcision was fear of pain during the procedure (56.3% among men aged >25 and 47% among men aged <25 years). Other barriers to VMMC included low risk perception (“I am not at risk of HIV infection,” “I am not promiscuous”) among both age groups; 14.6% (<25 years) and 13% (>25 years) respectively did not think they were at risk of HIV acquisition and 10.7% and 20.5% respectively did not think of themselves as being promiscuous. Lack of partner support for VMMC was cited by 14% of the interviewees from the older age group and only by 2.9% of the younger age group (p = 0.003). Perceived high costs were cited as a potential barrier by only 3.9% and 3.3% of the younger and older age group respectively (circumcision for HIV prevention is free in Zimbabwe).

**Table 5:** Barriers to VMMC by age among males aged 15–49 not willing to be circumcised.

Barrier	15–24 years (n = 103)	25–49 years (n = 215)	Total (n = 318)	Significance
Fear of pain	56.3	47.0	50.0	P = 0.119
I am not a risk of HIV	14.6	13.0	13.5	P = 0.707
I am not promiscuous	10.7	20.5	17.3	P = 0.031
My primary partner has not asked me	2.9	14.0	10.4	P = 0.003
I am worried about costs	3.9	3.3	3.5	P = 0.774
Other	37.9	38.6	38.4	P = 0.899

### Barriers to VMMC Uptake: Qualitative Findings

Qualitative findings suggested that fear of an HIV test is a major barrier to VMMC uptake. FGD participants mentioned that potential VMMC clients were opting not to be circumcised because they are required to first undertake an HIV test. *“They are saying before you are circumcised you must first get a blood test. That's what is chasing away most people,”* (uncircumcised man). Additionally, myths and misconceptions seem to be a significant deterrent. A few participants thought that VMMC was a potentially harmful procedure. *“I heard that they can even make the mistake of cutting your testes,”* (uncircumcised man). Yet others were concerned about the infertility that may arise as a result of circumcision. *“My major question is whether we will still be able to have children after circumcision,”* (uncircumcised men).

Partner refusal also came up as a major VMMC barrier among older, married men. A male partner described how his wife had confronted him and subsequently discouraged him from going for VMMC. *“She [wife] asked ‘why do you want to go for circumcision when you are already married? They say it offers prevention from HIV; where do you think the HIV will come from?’”* (uncircumcised man). Moreover, older men felt that the waiting time before resumption of sex post VMMC (six weeks) was too long. One man questioned: *“Do you think it is possible to sleep with your wife in the same blanket for six weeks without having sex?”* (uncircumcised man).

### Predictors of Uptake of VMMC

Segmentation analysis of the data collected in the population-based survey was used to predict uptake of VMMC. We identified availability, social support, perceived pain and self-efficacy as predictors of uptake of male circumcision (Table 6). Males reporting high perceived availability of VMMC services were more likely to be circumcised (OR = 2.32; p = 0.001). In addition, males reporting social support for VMMC from friends were also more likely to have been circumcised (OR = 3.01; p = 0.001). Males reporting perceived pain were least likely to be circumcised (OR = 0.71; p = 0.006). The strongest predictor of VMMC uptake was self-efficacy. Men with high levels of self-efficacy (one's belief that one can make the decision to go for VMMC) were eight times more likely of having been circumcised than men with low levels of self-efficacy (OR = 8.20; p = 0.042). Religion was a significant covariate. Male Christians were more likely to be circumcised compared to non-Christians (OR = 2.04; p = 0.014).

**Table 6:** Logistic regression for predictors of male circumcision uptake among adult males 15–49 years (outcome variable defined as males who were actually circumcised compared to those intending to be circumcised).

	AOR*/confidence interval	Significance
Availability	2.32 (1.67–3.22)	P = 0.001
Social support	3.01 (1.97–4.61)	P = 0.001
Self-efficacy	8.20 (1.08–62.04)	P = 0.042
Belief – MC procedure is painful	0.71 (0.56–0.91)	P = 0.006
Belief	1.31 (0.78–2.20)	P = 0.311
MC does not take too long to heal		
There are no negative side effects from the MC procedure		
MC is for responsible men who care about their health and their lives		
MC is beneficial only for men who are HIV- negative		
MC is safe		
**Population Characteristics**		
Education (secondary or higher vs. primary or lower)	0.39 (0.08–1.88)	P = 0.242
Age (15–24 vs. 25 and above)	1.00 (0.50–1.98)	P = 0.996
Socio-economic status	0.29 (0.72–1.10)	P = 0.289
Marital status (never and unmarried)	0.92 (0.50–1.87)	P = 0.924
Religion (Christian vs. non-Christian)	2.04 (1.16–3.61)	P = 0.014

*AORs = Adjusted Odds Ratio.

## Discussion

This research highlighted barriers and motivating factors to VMMC as well as predictors of VMMC uptake. In addition, it was useful in identifying how existing VMMC communication channels were used. It is encouraging to learn that knowledge of the protective effect of male circumcision seems to have increased since previous surveys [14], [15]. However, knowledge continues to be lower among females, who need to be specifically targeted by VMMC awareness campaigns. This is especially so given the fact that data suggest that women are likely to have considerable influence over their partner's decision to get circumcised, even if covert [7]. Additionally, our data suggested that while HIV is often cited as a reason for being circumcised, men are also motivated by non-HIV-related factors. This suggests that these non-HIV-related benefits should continue to be highlighted in demand-creation messages.

While there were no significant differences in VMMC knowledge among younger and older men, it was clear from the data that attitudes towards VMMC among the age groups differed and also, different factors motivated and deterred men of different ages. The findings suggest that demand-creation messages need to be specifically tailored for different age groups. Improved hygiene, perceived improved sexual performance and being an example to the community seemed to be motivating factors mainly cited by older age groups. Sexual performance and penile hygiene seem to be issues that mostly concern older men. Nonetheless, these motivating factors could be used in demand-creation campaigns especially targeting older age groups, while interventions using peer support seem to be effective in increasing positive attitudes towards VMMC particularly with younger age groups.

Participants indicated that they had mostly learned of VMMC from television and radio campaigns; these campaigns need to be intensified. New technological innovations such as mHealth (SMS messages) and social media need to be explored; these have been successfully used in other settings to promote VMMC and associated aspects [16]. The Zimbabwean teledensity of around 90% [17] bodes well for the use of such information dissemination mechanisms. These approaches need to be augmented by more participatory ones such as interpersonal communication initiatives that include the use of peers, community-based health care workers and community mobilizers, which have been successfully used to motivate men to take up VMMC in Kenya, Tanzania and South Africa [6]. These study findings suggest that these approaches are more effective when used to target adolescents and highlight the need for their intensification in both urban and rural settings.

The major barriers to VMMC included fear of pain, myths and misconceptions and lack of partner support. Fear of pain is a recurrent theme [7], [14], [18], [19]. Some of the fear is caused by awareness of traditional MC where pain is thought to be a pre-requisite for the procedure. Interventions to promote VMMC need to center on the fact that VMMC is a minor operation, which is not painful as it is performed under local anesthesia. Demand-creation needs to debunk myths and misconceptions around VMMC, such as male circumcision could potentially be harmful and may induce infertility. Since women play an important role in decision-making around VMMC, especially with older men in married relationships, and their potential support for VMMC is high [20], specific interventions need to be developed to increase their awareness and generate support for their partners and sons.

Of importance, a substantial proportion of men reported that they did not want to be circumcised because they were at low risk of HIV infection. Other surveys conducted in Zimbabwe and elsewhere suggest that people often underestimate their HIV risk and interventions to increase awareness of risk may serve to motivate men to undertake VMMC. Likewise, it is important to understand and address the myths and misconceptions deterring VMMC uptake, by context and age segment. In addition to knowledge about VMMC, the data demonstrate the need to strengthen self-efficacy, found to be the strongest predictor of VMMC uptake.

Findings from this and other studies have been used to enhance the Zimbabwe Ministry of Health and Child Care communication strategies. With support from PEPFAR through USAID and implemented by PSI, the mass media campaign was subsequently designed to position VMMC as a lifestyle choice rather than an HIV prevention method so as to increase acceptance of the service by both men and women, in addition to countering perceptions that the procedure only benefits “promiscuous” men. Thus, the campaign portrayed VMMC as a lifestyle choice for the “smart” man, one who is clean and elegant. The campaign sought to portray circumcised men as confident, outgoing, sexually appealing, and set to succeed in life. To address the predictive factor of social support, the program tailored messages for adolescents and young men by working with a local, popular artist who developed radio jingles and appeared on television, billboards and print materials promoting the hygiene aspect of VMMC. VMMC messages were also customized for women by highlighting MC's role in reducing the risk of cervical cancer, improving men's hygiene and enhancing men's sexual appeal.

Longer media formats such as radio and television programs were utilized to engage with the target audience and address various concerns, including those around pain and possible sexual dysfunction. Satisfied clients who had undergone VMMC appeared on scheduled radio and television programs to answer general questions, while doctors and nurses provided technical information about the procedure plus its associated health and HIV benefits. In addition, women whose partners had undergone the procedure discussed how they had supported the men. The women also described how they had themselves also benefitted from male circumcision. Mass media activities were complemented by community-level activities that reinforced the hygiene benefits of VMMC, addressed concerns around pain and myths and misconceptions. Younger and older men and women were trained at community level to promote the service among their peers through small group discussions and edutainment.

This exploration of motivators and barriers to VMMC through both qualitative and quantitative approaches yielded invaluable data. However, a potential limitation is that there was a three year gap between the conduct of the FGDs and the quantitative survey. Nonetheless, the difference in the time gap did not seem to have an effect on findings. By 2013, barriers to VMMC identified in 2010 had not changed significantly, hence the need to intensify and modify VMMC demand-creation strategies.

In conclusion, the qualitative and quantitative findings of this study highlighted major motivators and barriers to VMMC in Zimbabwe and informed the design of VMMC promotion activities. Continued campaigns and increased availability of services through scale-up will likely help towards increasing acceptability of the intervention.
